# A National Quality Improvement Program on Ultrasound Department in China: A Controlled Cohort Study of 1297 Public Hospitals

**DOI:** 10.3390/ijerph20010397

**Published:** 2022-12-27

**Authors:** Xixi Tao, Jianchu Li, Yang Gu, Li Ma, Wen Xu, Ruojiao Wang, Luying Gao, Rui Zhang, Hongyan Wang, Yuxin Jiang

**Affiliations:** 1 Department of Ultrasound, State Key Laboratory of Complex Severe and Rare Diseases, Peking Union Medical College Hospital, Chinese Academy of Medical Science and Peking Union Medical College, Beijing 100730, China; 2 National Ultrasound Quality Control Center, Beijing 100730, China

**Keywords:** quality improvement, ultrasound, quality indicator, workload, diagnostic accuracy

## Abstract

Providing high-quality medical services is of great importance in the imaging department, as there is a growing focus on personal health, and high-quality services can lead to improved patient outcomes. Many quality improvement (QI) programs with good guidance and fine measurement for improvement have been reported to be effective. In order to improve the quality of ultrasound departments in China, we conducted this study of a national quality improvement program. A total of 1297 public hospitals were included in this QI program on ultrasound departments in China from 2017 to 2019. The effect of this QI program was investigated, and potential factors, including hospital level and local economic development, were considered. The outcome indicators, the positive rate and diagnostic accuracy, were improved significantly between the two phases (positive rate, 2017 vs. 2019: 66.21% vs. 73.91%, *p* < 0.001; diagnostic accuracy, 2017 vs. 2019: 85.37% vs. 89.74%; *p* < 0.001). Additionally, they were improved in secondary and tertiary hospitals, with the improvement in secondary hospitals being greater. Notably, the enhancement of diagnostic accuracy in low-GDP provinces was almost 20%, which was more significant than the enhancement in high-GDP provinces. However, the important structural indicator, the doctor-to-patient ratio, decreased from 1.05:10,000 to 0.96:10,000 (*p* = 0.026). This study suggests that the national ultrasound QI program improved the outcome indicators, with secondary-level hospitals improving more than tertiary hospitals and low-GDP provinces improving more than high-GDP regions. Additionally, as there is a growing need for ultrasound examinations, more ultrasound doctors are needed in China.

## 1. Introduction

In recent years, with the growing focus on personal health, high-quality services have been expected in healthcare institutions. Quality has now become a popular theme in discussions concerning the healthcare system, contributing to the development of best practices that can lead to improved outcomes [1]. Quality in radiology was defined as ‘the extent to which the right procedure is performed in the right way, at the right time, and the correct interpretation is accurately and quickly communicated to the patient and referring physician’ [2]. Providing high-quality medical services in the imaging department is important for both clinical doctors and patients.

The meaning of quality improvement includes quality assurance programs for continuous quality improvement, processes to improve staff and patient safety, and procedures to improve clinical, technical, and diagnostic performance [3]. To date, a large number of quality improvement (QI) and system-based improvement initiatives in radiology have been implemented around the world, suggesting the global pursuit of improving quality and outcomes. The quality programs vary considerably in different countries and regions, but they can be classified by the common targets: radiology departments, doctors, equipment, and outcomes, and each target is not mutually exclusive [4]. It is suggested that QI programs with good guidance and a focus on measurement for improvement have an increased likelihood of success [5]. 

There are many QI programs reported to be effective in ultrasound or other medical imaging areas. Marriner et al. found that sonographers with a QI program of systematic quality checking will perform higher-quality echocardiograms and achieve high-quality patient care [6]. Yaqub et al. reported that QI interventions, including 22 sonographers with clinical auditing, feedback, and standardized scan protocols, helped to improve the completeness and quality of fetal anatomy screening [7]. Hui et al. improved the quality of pelvic ultrasound reports and reduced unnecessary imaging referrals for adnexal lesions by implementing a QI program aimed at providing appropriate descriptions and follow-up recommendations [8]. These studies provided a strong demonstration of the effect of the QI program. However, most of them focused on a specific group of diseases or clinical settings and only included a relatively small number of participants.

To date, there have been no reports of nationwide QI programs on ultrasound departments conducted in China, and there is limited empirical evidence assessing the quality of care in ultrasound departments in China. Given the huge population and growing development in China, the corresponding need for high-quality medical services is urged. Therefore, we conducted a national QI program with well-designed interventions, including the largest sample among investigations in ultrasound medicine in China, and assessed the effect of this QI program, comparing the quality indicators before and after the QI program’s implementation. Furthermore, the impacts of different hospital levels and local economic development were also considered when assessing the performance of the QI program. 

## 2. Methods

This study was a prospective cohort study approved by the Institutional Review Board of our institute. It was designed to evaluate the effect of a multifaceted national quality improvement program in ultrasound departments in China. This QI program was conducted by the China National Ultrasound Quality Control Center (China-NUQCC), which is an official national department that performs quality control measures in China. This QI program was carried out as an administrative instruction in the participating hospitals.

### 2.1. Intervention

The intervention of this QI program included the following three parts, and detailed information is shown in Supplementary Table S1.

Construction of a quality control team in each ultrasound department: The enrolled hospitals were required to construct a quality control team in each ultrasound department, with an experienced ultrasound doctor as the team leader. The quality control team was required to monitor the quality performance in the ultrasound department, collecting and analyzing the ultrasound quality indicators at regular intervals.

Training: The quality control team of each hospital received training from provincial and national ultrasound quality control centers using online and offline methods. The concerned subjects included ultrasound quality control indicators, standardized ultrasound exam protocols and reports, and other quality control materials.

Audits and feedback: Data of ultrasound quality control indicators were submitted online to China-NUQCC annually. The performance of ultrasound departments was audited by experts from provincial and national quality control centers using online or offline methods. Feedback was given to the sampled ultrasound department as the basis for follow-up adjustment.

### 2.2. Setting

This study was divided into two phases. The first phase was in 2017 when the quality control teams of participating hospitals were formed, and they were trained to record and analyze ultrasound quality data and input the data into the database of the National Clinical Improvement System (NCIS, https://ncisdc.medidata.cn/login.jsp, accessed on 20 July 2020), run by Department of the Medical Administration of the National Health Commission in China, and the access to data was approved by this government department. The quality data included the quality control indicators and other related information, and they were collected and submitted annually, and the data from 2017 were viewed as the baseline. In the second phase, the QI program for ultrasound was implemented. China-NUQCC obtained the data concerning quality control indicators from the database of NCIS. The enrolled hospitals were selected according to the following criteria: (1) the hospitals were public hospitals that had over 500 beds. (2) The ultrasound departments met the requirements of ultrasound departments in China. (Ultrasound departments are required to have ultrasound doctors who can carry out diagnostic ultrasounds independently. Ultrasound doctors are required to have both the certificate of doctor’s qualification and the license of medical practitioners in the field of medical imaging and nuclear medicine.) [9]. (3) The hospitals agreed to participate and were able to submit quality data.

### 2.3. Quality Control Indicators

Eight quality control indicators for ultrasound departments were used in this study, all of which were broadly and thoroughly discussed by experts in the field of ultrasound and medical quality control management [9]. Among the 8 quality control indicators, there were 5 structural indicators, 1 process indicator, and 2 outcome indicators. The definition and meaning of the quality indicators are shown in Table 1.

### 2.4. Statistical Analysis

We evaluated the differences in quality control indicators between the first (baseline) and second phases. The paired *t*-test and chi-squared test were used to assess the difference between the two phases according to the statistical types of data. Paired *t*-test was used to test the indicators, as the differences between the two phases are normally distributed, assessed by Q-Q plot, histogram, and Kolmogorov–Smirnov test. For each quality indicator, we also calculated median, upper and lower quartiles in enrolled hospitals, learning the variation between hospitals. In the subgroup analysis based on GDP per capita, the provinces were divided into low, medium, and high levels of GDP groups, according to the 2017 provincial GDP per capita from the 2017 China Statistical Yearbook. Analyses were performed using SPSS Statistics version 26.0 (IBM Corp., Armonk, NY, USA), and a *p*-value less than 0.05 was considered statistically significant. 

## 3. Results

### 3.1. Characteristics of Enrolled Hospitals

**Figure 1:**
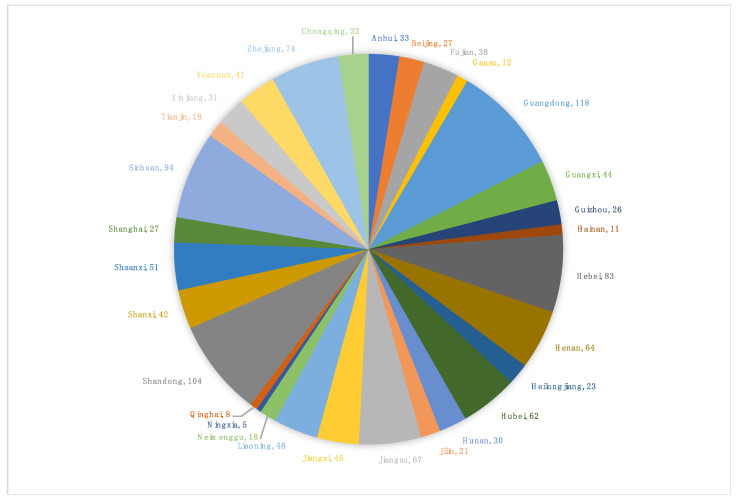
The composition of enrolled hospitals from 30 provinces in China.

There were a total of 1297 public hospitals enrolled in this QI program from 2017 to 2019 from 30 provinces in China. The composition of enrolled hospitals from each province is shown in Figure 1. In total, there were 21,406 doctors and 202,449,156 examinations in 2017, and 24,305 doctors and 253,481,288 examinations in 2019.

### 3.2. Effects of the QI Program on Ultrasound Departments

The doctor-to-patient ratio was 1.05:10,000 in 2017 and 0.96:10,000 in 2019 (*p* = 0.026). The examination-room-to-examination ratio was 0.68:10,000 in 2017 and 0.63:10,000 in 2019 (*p* < 0.001). The positive rate of ultrasound examinations and the accuracy of ultrasound diagnosis was found to be improved significantly between the two phases (positive rate, 2017 vs. 2019: 66.21 vs. 73.91, *p* < 0.001; diagnostic accuracy, 2017 vs. 2019: 85.37 vs. 89.74; *p* < 0.001). The other quality control indicators of the two phases demonstrated no significant differences, as shown in Table 2. The doctor-to-ultrasound-equipment ratios were very close (2017 vs. 2019: 1.35 vs. 1.34, *p* = 0.245). The average workload per doctor per working day was 37.83 in 2017 and 41.72 in 2019 (*p* = 0.846) (Table 2).

We also compared the variation in quality control indicators among enrolled hospitals (shown in Supplementary Table S2). There were large variations in most quality control indicators among enrolled hospitals. For example, the median doctor-to-patient ratio was 1.25:10,000 in 2017, while the lower quartile was 0.96:10,000, and the upper quartile was 1.71:10,000. Additionally, these variations were also greater in other structural quality indicators and average workload indicators.

### 3.3. Subgroup Analysis of Secondary and Tertiary Hospitals

There were 596 secondary hospitals and 701 tertiary hospitals in the enrolled hospitals. The doctor-to-patient ratio had decreased in the tertiary hospitals in 2019 (2017 vs. 2019: 1.09 vs. 0.88, *p* = 0.003), while this quality indicator suggested an opposite tendency in secondary hospitals (2017 vs. 2019: 0.98 vs. 1.29, *p* = 0.084). Similarly, the examination-room-to-examination ratio had decreased in tertiary hospitals from 2017 to 2019 (2017 vs. 2019: 0.72 vs. 0.60, *p* = 0.002) and increased in secondary hospitals (2017 vs. 2019: 0.59 vs. 0.79, *p* = 0.011). The two average workload quality indicators also showed different results in secondary and tertiary hospitals, as they both increased in tertiary hospitals and decreased in secondary hospitals, although some of the differences might not be statistically significant (Table 3).

Regarding the outcome quality indicators, both positive rate and diagnostic accuracy were found to be improved in secondary and tertiary hospitals, with the improvement in secondary hospitals being greater (Table 3). 

### 3.4. Subgroup Analysis Based on Provincial GDP per Capita

In order to further investigate the effect of the QI program, we also compared the hospitals from different provinces, grouped by different levels of GDP per capita. Ten provinces, including 567 hospitals, were regarded as high-GDP (GDP per capita ranging from 137,596 to 63,162 RMB); ten provinces, including 385 hospitals, were regarded as medium-GDP (GDP per capita ranging from 59,017 to 45,768 RMB); and ten provinces, including 345 hospitals, were regarded as low-GDP (GDP per capita ranging from 43,868 to 28,026 RMB). The doctor-to-patient ratio decreased from 2017 to 2019 in high-GDP provinces (2017 vs. 2019: 1.08:10,000 vs. 1.02:10,000, *p* = 0.006), and the examination-room-to-examination ratio also showed the same result in high-GDP and medium-GDP provinces. In regard to the average workload of outpatient services per hospital per working day, there were significant increases in high-GDP and low-GDP provinces. Lastly, the outcome indicators were found to be enhanced in provinces with different GDPs, except for diagnostic accuracy in medium-GDP provinces, with a slight decrease of 2.52%. Notably, the enhancement of diagnostic accuracy in low-GDP provinces was almost 20%, which was more significant than the enhancement in high-GDP provinces (Table 4). 

## 4. Discussion

We examined the effect of a 3-year quality improvement program in the ultrasound departments among 1297 secondary or tertiary public hospitals in China. We found that there was a significant improvement in the accuracy of ultrasound diagnosis, a key quality indicator in the ultrasound department, demonstrating the positive effect of this QI program. The systematic quality management program improved individual sonographers’ skills and their understanding of ultrasound examinations’ quality [6]. The enhancement of accuracy indicated that this QI program improved the doctors’ abilities to perform standardized ultrasound exam protocols and reach an accurate diagnosis, as well as the overall quality control procedure at the departmental level. 

The accuracy of ultrasound in China was 89.74% after the QI program in our study was implemented, suggesting that the diagnostic value of ultrasound in China is relatively high. To our knowledge, there is a lack of similar studies concerning quality improvement in the overall ultrasound diagnosis at the national level. Some studies have reported ultrasound accuracy in diagnosing a specific disease or a group of diseases, ranging from 65% to 97% [10,11]. A study conducted in an emergency department of a university hospital reported that the accuracy of ultrasound is 90% [12]. Additionally, the accuracy of ultrasound in the diagnosis of abdominal masses was reported to be about 87.4% in Nigeria [13]. Compared to these reported figures, the accuracy of ultrasound in China is relatively high, suggesting that ultrasound is a useful diagnostic tool in China.

Moreover, the positive rate of ultrasound examinations, reflecting the value and usefulness of ultrasound in clinical practice, was also found to be enhanced after the QI program. This indicates that the enforcement of standardized ultrasound exam protocols enables a more thorough and skilled scan, providing useful information for patient diagnosis and treatment plans. Admittedly, there are also other factors affecting the positive rate of ultrasound examinations, including aspects relating to clinicians and patients, as ultrasound examinations are safe, convenient, and more affordable compared to other radiology exams in China. To date, a wide range of positive rates in imaging studies have been reported, and no standards have been set. Additionally, many attempts to improve positive rates have been tried and validated [14,15]. Further studies are needed in order to set out a guideline or standard with regard to the acceptable utilization rates and positive rate, justifying the reasonable use of healthcare resources and better quality control, and detailed studies regarding different clinical settings are also needed. 

The improvement of both positive rate and accuracy in secondary hospitals was more prominent than in tertiary hospitals. This can be explained by the fact that tertiary hospitals had a higher level of faculty and facility in the first place, so the effect of this QI program was less prominent, while secondary hospitals could benefit from the program significantly. Therefore, the need to conduct a QI program in secondary hospitals is stronger and should be considered in future policy making.

The doctor-to-patient ratio, a structural quality control indicator for ultrasound departments, decreased from 2017 to 2019, suggesting that there were fewer ultrasound doctors compared to the increasing quantity of ultrasound examinations. As techniques in ultrasound departments have developed rapidly over the past few years, and the usage of ultrasound examinations has been applied more and more in clinical practice, not to mention the benefits of no radiation and relatively low cost, there is a growing need for ultrasound examinations in China. However, the number of ultrasound doctors has not seemed to be able to match the growing need lately since radiology residency training is arguably an intensive program, and it may take up to 8–10 years to train a qualified and skilled ultrasound doctor [16,17]. The problem of human resources in the ultrasound department should be paid attention to and addressed in order to provide sufficient and better medical services.

Moreover, this tendency was more prominent in tertiary hospitals, and the decreased examination-room-to-examination ratio may also suggest the growing need for ultrasound in tertiary hospitals, as this facility in hospitals was relatively stable compared to the fluctuation of workload. The remarkable increase in the average workload at both the doctor and hospital level in tertiary hospitals showed this tendency more directly, while the average workload per doctor per working day in secondary hospitals decreased by almost 10 examinations. The increasing population may contribute to the increasing need for ultrasound examinations, while the discrepancy in the workload of tertiary and secondary hospitals may be related to healthcare-seeking behavior in China. It has been noted that most patients tend to bypass primary care and acquire healthcare at higher-level hospitals, leading to overwhelming work stress in larger tertiary hospitals. In order to resolve this problem, the National Hierarchical Medical System (NHMS) is being implemented by the Chinese government, supporting the development of primary care medical institutions [18,19]. Many relevant policies, including the national general practitioner system and the two-way referral system, have also been launched by the Chinese government. Hopefully, medical resources will be more balanced and utilized more reasonably in the future.

Waiting time is a critical factor affecting patients’ satisfaction and the efficiency of medical services, and this study provides information on this aspect [20]. Although there are no significant differences in the average waiting times for an inpatient appointment between 2017 and 2019, the average waiting time was less than 2 days, suggesting ultrasounds were performed efficiently and in a timely manner in China, as patients can experience significant waiting times for various diagnostic technologies in other countries, with the time being up to 2–20 weeks for non-urgent situations [21,22].

The subgroup analysis showed a significant increase in the average workload of outpatient services in both high- and low-GDP provinces, and the increase in high-GDP provinces was more prominent. Previous studies have shown that there is a gap in the spatial distribution of medical resources in China, and resources are concentrated in economically developed provinces, despite significant improvements in health services in each province [23]. The imbalance of medical resources contributes to the flow of patients between provinces, increasing the workload in resource-centered provinces. Furthermore, the high workload makes it difficult for doctors to spend sufficient time with patients, which may affect the quality of care, patient safety, and the doctor–patient relationship [24]. Effective health and policy interventions are needed to enhance the development in less-developed provinces and narrow the gap between provinces, providing better and sufficient health services for people in each province [25].

Outcome indicators provide important audits for hospitals and relevant government departments, prompting the diagnosis of quality gaps and the identification of the root cause [26]. The positive rate of ultrasound scans increased to a similar extent in high-, medium-, and low-GDP provinces, suggesting similar results of the QI program in different provinces. As for the accuracy of ultrasound diagnosis, there was an obvious increase in low-GDP provinces, while the increase in the high-GDP province was very slight. In our study, the diagnostic accuracy in 2017 was relatively low in low-GDP provinces, which may be related to their financial disadvantages, the lack of high-level human resources and graduate medical education. The obvious increase in accuracy suggested the positive effect and the necessity of this QI program in low-GDP provinces, as well as the need for policy and educational support in low-GDP provinces. 

To our knowledge, this study is the first nationwide study of ultrasound departments in China that analyzes data between 2017 and 2019, including a large number of hospitals from 30 provinces. As there is limited empirical evidence assessing the quality of ultrasound in China’s health system, our work reflected the national policy on ultrasound quality control and the impact factors in China, providing important insights into the quality of ultrasound departments in China. This study included reproducible metrics, such as waiting time, diagnostic accuracy, and the positive rate of scans, reflecting the performance of the ultrasound profession at the national level and allowing meaningful data points to guide future improvement [27]. This QI program was effectively implemented by the administration of China-NUQCC with great effort, and a hierarchical ultrasound quality control system was built, ranging from the national to the provincial, regional, and hospital levels. Hence, we can achieve effective quality management and collect quality indicator data on a national scale.

There were some limitations in this study. First, this study is not a randomized controlled study, so there may be some confounding factors. For example, the majority of the enrolled hospitals are from high-GDP provinces, which may be related to their better-developed information system and higher motivation to attend the QI program. Second, the analysis of outcome indicators did not separate different sources of patients, as ultrasound examination is used in different settings in outpatient, inpatient, or emergency departments. However, we are able to provide quality information at the overall level, and further study is needed to obtain detailed information. Third, this study analyzed the effect of the QI program over a 3-year period, which may not be long enough for some interventions to work and produce a noticeable effect. Furthermore, the Hawthorne effect should be acknowledged, as subjects being studied may improve or modify their behavior [28]. Additionally, quantitative metrics may pressure the subjects to pay inordinate attention to making statistics look good [29].

## 5. Conclusions

In this nationwide 3-year QI program, we observed improvement in the outcome indicators of diagnostic accuracy and positive rate, with secondary-level hospitals and hospitals in low-GDP provinces benefiting more. Additionally, the number of ultrasound doctors may not be comparable to the growing need for ultrasound examinations in this 3-year period. The policy should be made to support the development of hospitals of secondary-level or from less-developed areas and address the problem of insufficient human resources in ultrasound departments in the future. Hopefully, our findings will contribute to the development of guidelines and standards to ensure rational and effective quality management to improve patient-centered outcomes. 

## Figures and Tables

**Table 1:** Definition and meaning of the quality control indicators.

Quality Control Indicators	Definition and Formula
Doctor-to-patient ratio (1:10,000)	Definition: the ratio of ultrasound doctors to ultrasound examinations completed during the same period
	Formula: (ultrasound doctors)/(ultrasound examinations completed during the same period) × 10,000
Examination-room-to-examination ratio (1:10,000)	Definition: the ratio of ultrasound examination rooms to ultrasound examinations completed during the same period
	Formula: (ultrasound examination rooms)/(ultrasound examinations completed during the same period) × 10,000
Doctor-to-ultrasound-equipment ratio	Definition: the ratio of ultrasound doctors to ultrasound equipment during the same period
	Formula: (ultrasound doctors)/(ultrasound equipment during the same period)
Average workload per doctor per working day	Definition: the average number of ultrasound examinations (including exams of inpatient, outpatient, emergency room) completed by each ultrasound doctor per working day
	Formula: (ultrasound examinations completed in a year)/(ultrasound doctors during the same period)/(working days during the same period)
Average workload of outpatient service per hospital per working day	Definition: the average number of ultrasound examinations of outpatient service completed by each hospital per working day
	Formula: (ultrasound examinations of outpatient service completed in a year)/(working days during the same period)
Average waiting days for inpatient appointment	Definition: the average number of days spent waiting for inpatient ultrasound examination from being ordered to being conducted
	Formula: (waiting days of all inpatient ultrasound examinations)/(inpatient ultrasound examinations during the same period)
Positive rate of ultrasound examinations (%)	Definition: the proportion of ultrasound examinations with any positive finding among all ultrasound examinations during the same period
	Formula: (ultrasound examinations with positive findings)/(ultrasound examinations during the same period)
Accuracy of ultrasound diagnosis (%)	Definition: the proportion of ultrasound examinations with accurate diagnosis compared to the result of pathology or clinical diagnosis among all randomly chosen ultrasound examinations with results of pathology or clinical diagnosis (20 ultrasound examinations per doctor were required to be inspected at least)
	Formula: (ultrasound examinations with accurate diagnosis compared to the result of pathology or clinical diagnosis)/(all randomly chosen ultrasound examinations with the result of pathology or clinical diagnosis)

**Table 2:** Performance on quality control indicators in enrolled hospitals.

Quality Control Indicators	2017	2019	*p*-Value
Doctor-to-patient ratio (1:10,000)	1.05	0.96	0.026
Examination-room-to-examination ratio (1:10,000)	0.68	0.63	<0.001
Doctor-to-ultrasound-equipment ratio	1.35	1.34	0.245
Average workload per doctor per working day	37.83	41.72	0.846
Average workload of outpatient service per hospital per working day	345.59	388.17	0.472
Average waiting days for inpatient appointment	1.39	1.34	0.473
Positive rate of ultrasound examination (%)	66.21	73.91	<0.001
Accuracy of ultrasound diagnosis (%)	85.37	89.74	<0.001

**Table 3:** Performance in quality control indicators in enrolled hospitals classified by hospital grade.

Quality Control Indicators	2017	2019	*p*-Value
Doctor-to-patient ratio			
Secondary hospital	0.98	1.29	0.084
Tertiary hospital	1.09	0.88	0.003
Examination-room-to-examination ratio			
Secondary hospital	0.59	0.79	0.011
Tertiary hospital	0.72	0.60	0.002
Doctor-to-ultrasound-equipment ratio			
Secondary hospital	1.51	1.48	0.923
Tertiary hospital	1.31	1.30	0.013
Average workload per doctor per working day			
Secondary hospital	40.83	30.91	<0.001
Tertiary hospital	36.76	45.42	<0.001
Average workload of outpatient service per hospital per working day			
Secondary hospital	262.00	176.18	0.403
Tertiary hospital	416.84	568.40	0.021
Average waiting days for inpatient appointment			
Secondary hospital	1.37	1.22	0.443
Tertiary hospital	1.40	1.40	0.791
Positive rate of ultrasound examinations			
Secondary hospital	63.39	73.76	<0.001
Tertiary hospital	68.57	74.03	<0.001
Accuracy of ultrasound diagnosis			
Secondary hospital	74.85	87.82	<0.001
Tertiary hospital	89.70	92.39	<0.001

**Table 4:** Performance in quality control indicators of high-GDP, medium-GDP, and low-GDP provinces.

Quality Control Indicators	2017	2019	*p*-Value
Doctor-to-patient ratio			
High-GDP	1.08	1.02	0.006
Medium-GDP	0.92	0.76	0.114
Low-GDP	1.26	1.20	0.167
Examination-room-to-examination ratio			
High-GDP	0.73	0.70	0.006
Medium-GDP	0.58	0.50	0.025
Low-GDP	0.77	0.73	0.151
Doctor-to-ultrasound-equipment ratio			
High-GDP	1.29	1.28	0.098
Medium-GDP	1.41	1.36	0.378
Low-GDP	1.44	1.45	0.689
Average workload per doctor per working day			
High-GDP	37.20	39.40	0.150
Medium-GDP	43.55	52.32	0.791
Low-GDP	31.86	33.44	0.761
Average workload of outpatient service per hospital per working day			
High-GDP	368.41	431.38	<0.001
Medium-GDP	424.62	437.51	0.940
Low-GDP	220.36	262.07	0.001
Average waiting days for inpatient appointment			
High-GDP	1.45	1.44	0.282
Medium-GDP	1.16	1.41	0.091
Low-GDP	1.57	1.11	0.195
Positive rate of ultrasound scans			
High-GDP	64.64	72.76	<0.001
Medium-GDP	68.06	76.53	<0.001
Low-GDP	66.77	72.89	<0.001
Accuracy of ultrasound diagnosis			
High-GDP	91.81	92.17	<0.001
Medium-GDP	92.79	90.27	<0.001
Low-GDP	67.95	87.54	<0.001

## Data Availability

Datasets used in this study are not publicly available due to embedded legal policies. Data were provided by the Department of Medical Administration, National Health Commission of China. Access to datasets is granted to allowable bodies upon agreement from involved institutions. Requests can be made to the director of the service.

## Supplementary Materials

The following supporting information can be downloaded at: https://www.mdpi.com/article/10.3390/ijerph20010397/s1, Table S1: Interventions of QI program; Table S2: Variation in quality control indicators among enrolled hospitals. Reference [30] is cited in the supplementary materials.
