# Supplementary material for: A National Quality Improvement Program on Ultrasound Department in China: A Controlled Cohort Study of 1297 Public Hospitals
Source: Int J Environ Res Public Health. 2022 Dec 27;20(1):397. doi: 10.3390/ijerph20010397 (PMC9819884; doi:10.3390/ijerph20010397)
Supplement: Supplementary file 1 [file ijerph-20-00397-s001.zip › ijerph-2037748-supplementary.pdf]

International Journal of
Environmental Research and Public Health

MDPI

Table S1. Interventions of QI program.

Interventions	Contents
Construction of quality control team in each ultrasound department	A quality control team is required to be built in each ultrasound department, with an experienced ultrasound doctor as the team leader, who must be a chief or associate chief doctor (the highest two professional titles in medical system in China) and has at least 10 years of experience in ultrasound practice, and a doctor as the special contact submitting and reporting quality control data on the internet.<br>Quarterly departmental quality control meeting: Quality review and documentation are performed quarterly, randomly selected examinations are evaluated in the following criteria. (1) image quality and completeness, (2) report quality and completeness, (3) adherence to protocols, (4) accuracy of diagnosis (compared to the result of pathology or clinical diagnosis). The results of all the reviews and misdiagnosed cases are presented for discussion and improvement of examination quality.<br>Deming cycle (Plan-Do-Check-Action (PDCA)) method to improve processes related to the outcome quality indicators in ultrasound department.
Training	The quality control teams receive training on the definition and meaning of ultrasound quality control indicators on the website of China-NUQCC (www.nuqcc.cn).<br>The annual national conference of ultrasound quality control in China: the ultrasound doctors from all the participated hospitals attend the conference online or offline. The conference provides training on quality improvement methods, standardized ultrasound examination protocols, international guidelines, and other quality control topics.<br>Quarterly departmental quality control meeting: all the members will learn the quality control materials.<br>Web-based training: online courses about quality control and standardized ultrasound examination of different diseases or systems on the website of China-NUQCC (www.nuqcc.cn).<br>And these training are lectured by doctors of ultrasound and other professions who are experienced in medical quality control, such as experts from other national or provincial quality control centers.<br>Update guidelines and expert consensus on ultrasound quality controls and ultrasound diagnosis by the China-NUQCC or provincial quality control centers.<br>Training materials: definition and meaning of quality control indicators, quality control management standards of medical ultrasound [1,2].
Audits and feedbacks	Each quality control team submits quality control data of their department online to China-NUQCC annually.<br>Each quality control team submits at least 20 cases quarterly, including ultrasound images, reports, patient history, pathologic or other exam results. The images and reports will be audited by experts who are attending, associate chief or chief doctor of ul-

	trasound medicine, have a minimum of 10 years of experience in ultrasound practice, and core members from national or provincial quality control centers and the feedback will be given to the local teams.<br>A hotline is provided by China-NUQCC, offering point-to-point communication about quality control indicators and other quality management issues.<br>On-site supervision by national and provincial centers: randomly selected ultrasound department are supervised and scored based on the quality control team construction, the management of human resources, equipment, examination rooms, and work protocols, PDCA cycle for improving quality outcome indicators, and all the related documentation.

References

1. China National Ultrasound Quality Control Center; Ultrasound Medicine Branch of Chinese Medical Association. Expert Consensus on Quality Control Indicators for Medical Ultrasound (2018). *Chin. J. Ultrason.* **2018**, *27*, 921–923.
2. China National Ultrasound Quality Control Center; Beijing Ultrasound Quality Control Center. *Quality Control Management Standards of Medical Ultrasound*; People's Medical Publishing House: Beijing, China, 2017.

Table S2. Variation of quality control indicators among enrolled hospitals.

Quality Control Indicators	2017	2019
Doctor-to-patient ratio(1:10,000)	1.25 (0.96–1.71)	1.23 (0.92–1.65)
Examination-room-to-examination ratio(1:10,000)	0.78 (0.6–1.06)	0.77 (0.6–1.02)
Doctor-to-ultrasound-equipment ratio	1.40 (1.16–1.75)	1.38 (1.17–1.68)
Average workload per doctor per working day	31.98 (23.41–41.61)	32.48 (24.17–43.26)
Average workload of outpatient service per hospital per working day	207.53 (110.19–379.25)	242.04 (127.08–440.13)
Average waiting days for inpatient appointment	1.00 (0–2.00)	1.00 (0–1.53)
Positive rate of ultrasound examinations (%)	72.00 (58.33–82.33)	76.33 (67.67–84)
Accuracy of ultrasound diagnosis (%)	91.88 (85.33–95.65)	92.31 (86.3–95.97)

Data are presented as median values, 25th and 75th interquartile ranges.
